# Dementia care coordinator service in Kent and Medway: a realist evaluation protocol

**DOI:** 10.3399/BJGPO.2023.0098

**Published:** 2023-11-15

**Authors:** Ruth Abrams, Heather Gage, Jill Maben, Wendy Grosvenor, Kath Sykes, Morro Touray

**Affiliations:** 1 University of Surrey, Kate Granger Building, Surrey Research Park, Guildford, UK; 2 Department of Clinical and Experimental Medicine, University of Surrey, Surrey Health Economics Centre, Leggett Building, Guildford, UK; 3 Applied Research Collaboration/Academic Health Science Network, Beehive, Gatwick, UK

**Keywords:** dementia, primary health care, general practice, role implementation, service design, service evaluation, realist approach

## Abstract

**Background:**

Dementia care is a key priority for both NHS England and the UK government. National guidelines highlight the importance of care coordination to address the challenges people living with dementia and their carers can encounter when trying to access the health and care system. To counter these challenges, Kent and Medway Integrated Care Board (ICB) have recently implemented a proactive dementia care coordinator (DCC) service to support people with dementia and their carers from pre-diagnosis to end-of-life care.

**Aim:**

To understand how the DCC service works (or does not work), for whom, and in what circumstances. The findings will inform service development and future investment decisions.

**Design & setting:**

This study will use a realist approach to evaluate the DCC service in Kent and Medway ICB, south-east England, which has a population of 1.9 million, comprising 42 primary care networks (PCNs; groups of general practices) each having a DCC.

**Method:**

An initial programme theory will be developed from existing literature, and in collaboration with stakeholders. Mixed methods, including questionnaires to DCCs, service provider metrics, and qualitative interviews, will be used to collect data on service provider and service user experiences. Interpretive comparative analysis and narrative synthesis, including evaluation of service costs against outcomes, will produce a refined final programme theory.

**Results:**

The results from this project will produce evidence-based recommendations to help improve service delivery and possible service expansion.

**Conclusion:**

This protocol describes a realist evaluation designed to investigate the recently implemented DCC service in Kent and Medway ICB.

## How this fits in

Dementia services in primary care are undergoing a period of transformation. In Kent and Medway, a recent implemented service has been that of the DCC, a proactive role to help people living with dementia and carers navigate the care system. Yet challenges exist when implementing a new role and new service into an existing primary care organisation. This realist evaluation will explore what works, for whom, how, why, and under what circumstances. Findings will provide evidence capable of informing future decisions about the DCC service including how the service can be successfully improved and expanded, where appropriate.

## Introduction

Dementia care is a key priority for both NHS England and the UK government. Improving diagnosis rates and ensuring people have access to high-quality support both before and after diagnosis has been central to two Prime Minister Challenges on Dementia (2012–2015 and 2020),^1^ and the NHS Long Term Plan.^2^ The National Institute for Health and Care Excellence (NICE) guidelines on dementia highlight that people living with dementia should have a single-named health or social care professional, in order to improve access to and coordination of their care.^3^ Consequently, dementia services are undergoing a period of transformation.^4^

In response to the national initiatives, the Kent and Medway ICB conceived and jointly commissioned a DCC service across the region. Kent and Medway, in the south-east corner of England, has a population of 1.868 million. The ICB area is divided into 42 PCNs, that is, local groupings of GPs serving between 30 000 and 50 000 people each, in four health and care partnership areas. The DCC service is proactive and intended to support people with dementia and their carers to navigate the care system from pre-diagnosis to end of life, including bereavement support, if needed. It has been designed to address issues of local importance, namely low dementia diagnosis rates and fragmented post-diagnosis support. Following a small pilot project in eight PCNs in 2021, the ICB has funded new DCC roles in all 42 PCNs, starting from April 2022, for 2 years.

The purpose of this study is to use realist methods to evaluate the new service. The aim is to understand how the DCC service works (or does not work), for whom, and in what circumstances. The findings will inform service development and future investment decisions.

The research questions are:

1. What are the experiences of stakeholders (including people with dementia and carers) of the DCC service?
2. How has the implementation of DCCs produced intended outcomes (increased diagnosis rates, improved community support, reduced unplanned hospital admissions) and any unintended outcomes?
3. What are the contextual factors that impact on if or how the service produces outcomes?
4. What are the resource or cost implications of the DCC service?

## Method

### Study design

**Figure 1.**
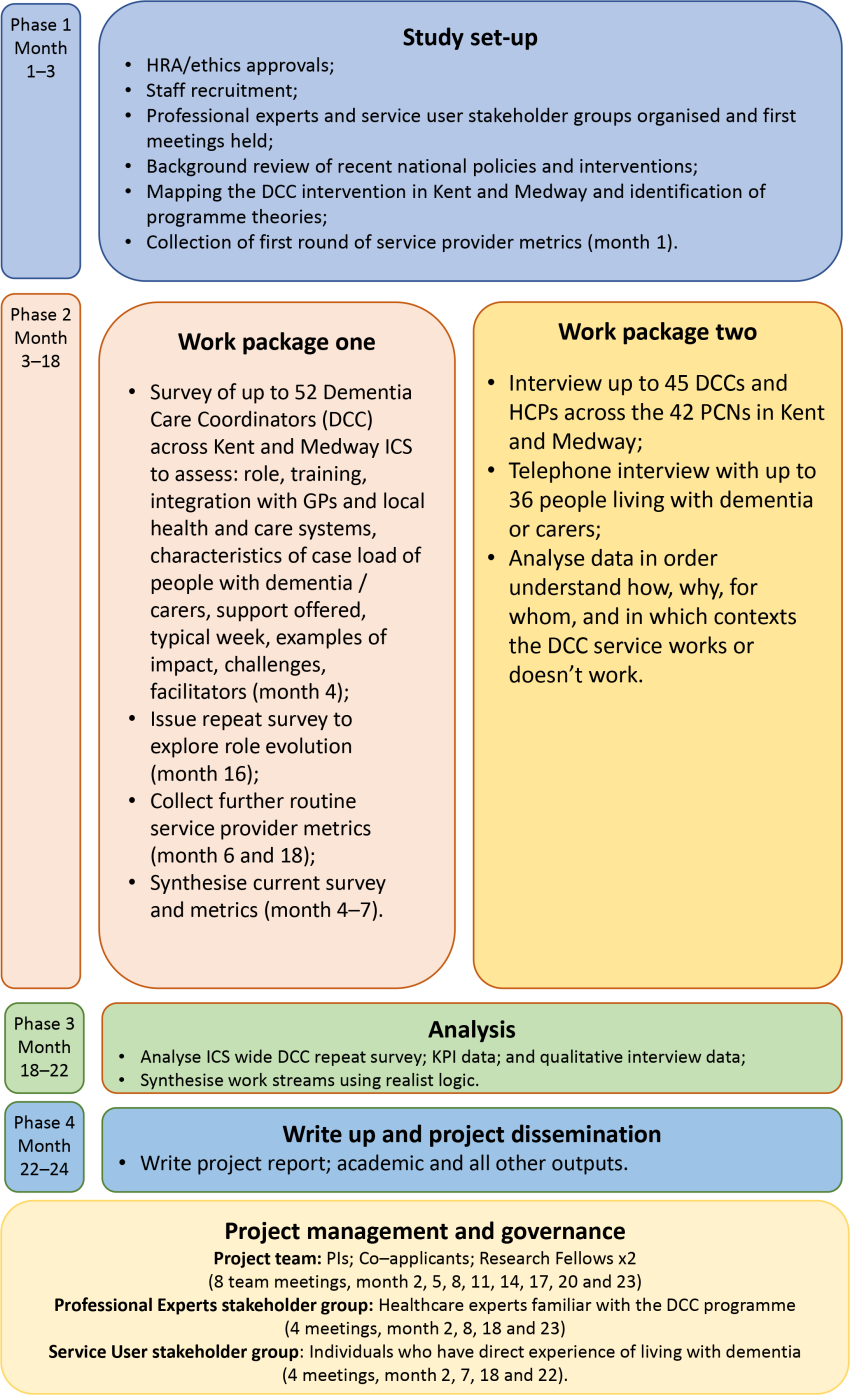
Study flowchart. DCC = dementia care coordinator. HCPs = healthcare professionals. HRA = Health Research Authority. ICS = integrated care system. KPI = key performance indicators

Realist evaluation goes beyond the simple question of whether a programme, intervention, or service works or not, to understand how, why, for whom, and in what circumstances.^5^ Realist evaluation is concerned with understanding causal mechanisms and the conditions under which they are activated to produce specific outcomes. This approach is also consistent with the new Medical Research Council framework for evaluating complex interventions.^6^ See Figure 1 for the study flowchart.

### Stakeholder advisory groups

Two stakeholder groups were established at the outset to advise the research team. Each will meet four times during the 24-month study period.

1. A service user and carer group will comprise experts by experience, namely two people living with dementia and four carers. They will ensure that the study team remains sensitive to the needs of those receiving the service. Their role is to advise on aspects relating to those with dementia and their carers, including the following: recruitment of participants; the interview topic guide; interpretation of findings; construction of recommendations; and dissemination activity. Members of the group will be remunerated for their time.
2. A professional healthcare expert group, comprising dementia healthcare professionals from Kent and Medway with an interest and involvement in the DCC service. This group will help the evaluation deliver on its core aims and provide support as needed. Members will also assist with the design of the surveys, refining the interview topic guide for professionals, and the identification and dissemination of key messages.

### Setting

Kent and Medway is varied geographically, containing urban, rural, and coastal areas. Its local authorities span all deprivation deciles, and there are pockets of high ethnic diversity in the north of the county.^7^ It is estimated that there are approximately 27 000 people currently living with dementia in Kent and Medway, and this number is expected to increase to 38 000 by 2030.^8^

### Intervention

DCCs are flexible and proactive. They are employed by one or other of the two commissioned providers in the region, sit within primary care, and act as a liaison within the community. They work in conjunction with a multidisciplinary team (MDT) of professionals across the health and social care system. Their role is to assess the needs of people living with dementia, and identify and agree person-centred outcomes to improve quality of life. DCCs receive referrals from multiple sources including GPs, memory services, social services, and self-referral. They aim to make a first contact call for an initial assessment within 2 days of referral and plan for a home visit to undertake a comprehensive assessment within 7 days. Once a plan of action has been established, the case is temporarily closed and a follow-up is scheduled every 3 months, in order to manage caseload. This is a non-clinical role. DCCs do not necessarily have a background in dementia care, nor do they need qualifications; however, they are provided with training by the service providers.

### Research plan

#### Phase 1: Background data collection

1.1 An initial programme theory (that is, a complex mapping of how the service is designed to work) will be developed at the outset of the project, generated from commissioning documents (for example, existing case studies from the service), data collected from the pilot project, and preliminary conversations with the stakeholder groups. Recent evidence will also be identified on dedicated dementia support interventions in UK and comparable healthcare systems. Searches will be conducted in MEDLINE, CINHAL (Cumulative Index of Nursing and Allied Health Literature), and PsychINFO, will focus on reviews of reviews, and will provide a narrative synthesis. Search terms used will include, for example, dementia, dementia-friendly intervention, dementia-friendly communities, social prescribing, care navigation, social participation, caregiving in dementia, role implementation, role integration, dementia coordinator, dementia care coordinator, dementia support coordinator, among others. Following the realist methodology, data collection in Phase 2 will seek to refine or refute the programme’s intentions, leading to a set of evidence-based implications for practice to help improve service design and delivery.

#### Phase 2: Evaluation of DCC service

The service will be evaluated in two work packages, using quantitative and qualitative methods, area-wide across the 42 PCNs in Kent and Medway.

#### 2.1 Work package one

1. Repeat online surveys (using Qualtrics) will be sent to all DCCs across the 42 PCNs, at months 3 (initial) and 16 (follow-up) to provide an overview of the service implementation and evolution. The surveys will include a mixture of qualitative and quantitative questions, developed by the project team and piloted before distribution. Questions will cover the following: DCC’s prior experience in dementia care; training and support received; and details of what the role involves through a description of a typical day (categorised as time spent on emails and phone messages, home visits, phone calls with clients, making records and referrals, attending MDT meetings, supervisions, breaks).
2. Organisational metrics, including process and outcome indicators, will be obtained in months 1, 6, and 18 from routine PCN-level data collected by the service provider organisations. This will include, for example, the number of people with dementia referred to the service, and source of referral, DCC activity (caseloads, numbers referred for pre-diagnosis discussion, progression to diagnosis, completed post-diagnosis episodes and support provided, quality of life and carer strain outcome indicators, service satisfaction measures).

#### 2.2 Work package two

1. Online interviews (*n* = 45) will be conducted with service providers (for example, DCCs) and other healthcare professionals (for example, GPs, nurses) across Kent and Medway to explore their perspectives and experiences of service delivery in order to refine and consolidate the programme theory.^9^ An interview topic guide will be created in collaboration with the professional expert group and will ask questions about how DCCs and other healthcare professionals work together.
2. Online or telephone interviews (*n* = 36) will be conducted with service users (for example, people with dementia or carers) to explore their perceptions and experiences of the DCC service. The authors will work with the DCCs interviewed to help identify and invite participants from varied socioeconomic groups. An interview topic guide will be created in collaboration with the service user and carer group. Only those with the capacity to consent will be invited to participate.

Table 1 shows the full inclusion and exclusion criteria for both professional and service user participants

**Table 1.** Study eligibility

Group	Inclusion criteria
Professional experts	DCCs and HCPs who have significant involvement in the DCC service in Kent and Medway ICB Aged ≥18 years Fluent in English language (the use of translation services are beyond the resources of this project) Any gender, ethnic group, or socioeconomic status Have an internet connection, a device (phone, tablet, laptop) with internet access and broadband width, or a landline or mobile phone
People with dementia	Adults living with dementia who have the capacity to consent Adults living with dementia who are registered at a GP practice in Kent and Medway and used the DCC programme Fluent in English language (the use of translation services are beyond the resources of this project) Cognitively able to respond to short question and answers Cognitively able to consent to participating in a short telephone interview Any gender, ethnic group, or socioeconomic status Have an internet connection, a device (phone, tablet, laptop) with internet access and broadband width, or a landline or mobile phone
Carers	Adults caring for a person living with dementia (aged ≥18 years) Adults caring for a person living with dementia who are registered at a GP practice in Kent and Medway and used the DCC programme Fluent in English language (the use of translation services are beyond the resources of this project) Consent to participating in a short interview Any gender, ethnic group, or socioeconomic status Have an internet connection, a device (phone, tablet, laptop) with internet access, or a landline or mobile phone
Exclusion criteria Outside of stated age range Outside stated of location Unable to access telephone or internet Unable to consent or respond to questions cognitively or in English	

DCCs = dementia care coordinators. HCPs = healthcare professionals. ICB = integrated care board.

#### Phase 3: Analysis and write-up

Findings from across the entire project will be analysed separately and then synthesised using a realist logic approach, consolidating data to refine the programme theory (following the Realist and Meta-Review Evidence Synthesis: Evolving Standards; RAMESES II).^10^

Quantitative data from the DCC survey and organisational metrics will be analysed descriptively and qualitative data will be analysed thematically to provide a comparative report of the implementation of the DCC service across the 42 PCNs and how it evolves over 18 months. Variability will be explored with reference to routine data relating to the social, economic, demographic, and geographical characteristics of the PCNs drawn from available sources (for example, Public Health England’s general practice profiles), and the ICB’s own information systems. Context-mechanism-outcome configurations will be constructed from this data and cross-compared with the initial programme theory and data from the qualitative interviews.

Qualitative interview data will be thematically analysed concurrently with data collection. Coding will be deductive (informed by existing theory), inductive (from the data collected), and retroductive (inferences based on interpretations of the data collected about underlying causal mechanisms). Interpretive cross-case comparison will be used to understand and explain how and why observed outcomes have occurred, for example, by making comparisons between different sources of data, to understand how context has influenced reported findings. Data to inform the interpretation of the relationships between contexts, mechanisms, and outcomes will be sought not just within the same interviews, but across interviews.

An indicative health economic analysis will investigate the resource implications and costs of the DCC service at the aggregate service-provision level. A narrative evaluation of costs against outcomes in a costs consequences framework incorporating all relevant outcomes for stakeholders will be presented as data permit.

Write-up and dissemination activities will take place across months 22–24.

### Ethical considerations

Written consent will be sought and obtained before participation. All participants will have the right to withdraw. The study will only include people living with dementia who have the capacity to consent. The study has a process in place if at the time of interview a participant appears confused, distressed, or unable to continue. The identity of all participants will be protected with pseudonyms.

## Discussion

### Summary

Dementia is a national priority and policies require local commissioners to implement support services for those affected. However, evidence suggests that the implementation of new roles and new services into existing infrastructure presents challenges.^11,12^ Evaluating the new DCC service in Kent and Medway and building a programme theory from multiple stakeholder perspectives, including both service providers and service users, will provide insight into how commissioners can support local need by identifying what is working well, what is not working well, and, crucially, why. The findings from this evaluation will inform decision making about the future structure of services both in Kent and Medway and more widely. It will contribute to the evidence base in two key areas, including: (1) service or role transformation; and (2) dementia care. This is especially important at a time when primary care is seeing ongoing disruptions to service delivery, challenges to patient access, and a workforce crisis.

### Strengths and limitations

A strength of this project is its realist methodological approach, which will go beyond exploring simply what works, to identify how, why, and under what circumstances. The research is constrained by its limited resources, which prevents use of interpreters and inclusion of those who do not have English as their first language. Resource constraints also restrict primary data collection, the feasible sample size, and the overall scope of the evaluation. There may also be challenges to conducting remote interviews (that is, online or telephone) with people living with dementia. However, this was felt to be a safe and appropriate approach to limiting distress in this participant group.

### Implications for research and practice

There are a number of other projects exploring the implementation of dementia-specific support in primary care including (but not limited to) for example D-PACT.^13^ This indicates a timeliness and necessity to this work. Ensuring results from a number of studies exploring dementia services converge, may help to strengthen insight into both service improvement and service expansion in dementia care and service transformation, so that service users and service providers wanting to support them can benefit.

## Data

Data can be made available upon reasonable request from the corresponding author.
